# Using 16S rRNA gene as marker to detect unknown bacteria in microbial communities

**DOI:** 10.1186/s12859-017-1901-8

**Published:** 2017-12-28

**Authors:** Quang Tran, Diem-Trang Pham, Vinhthuy Phan

**Affiliations:** Department of Computer Science, University of Memphis, Memphis, 38152 TN USA

**Keywords:** Metagenomics, Bacteria detection, NGS analysis

## Abstract

**Background:**

Quantification and identification of microbial genomes based on next-generation sequencing data is a challenging problem in metagenomics. Although current methods have mostly focused on analyzing bacteria whose genomes have been sequenced, such analyses are, however, complicated by the presence of unknown bacteria or bacteria whose genomes have not been sequence.

**Results:**

We propose a method for detecting unknown bacteria in environmental samples. Our approach is unique in its utilization of short reads only from 16S rRNA genes, not from entire genomes. We show that short reads from 16S rRNA genes retain sufficient information for detecting unknown bacteria in oral microbial communities.

**Conclusion:**

In our experimentation with bacterial genomes from the Human Oral Microbiome Database, we found that this method made accurate and robust predictions at different read coverages and percentages of unknown bacteria. Advantages of this approach include not only a reduction in experimental and computational costs but also a potentially high accuracy across environmental samples due to the strong conservation of the 16S rRNA gene.

## Background

In these profiling microbial communities, the main objective is to identify which bacteria and how much they are present in the environments. Most microbial profiling methods focus on the identification and quantification of bacteria with already sequenced genomes. Further, most methods utilize information obtained from entire genomes. Homology-based methods such as [1–4] classify sequences by detecting homology in reads belonging to either an entire genome or only a small set of marker genes. Composition-based methods generally use conserved compositional features of genomes for classification and as such they utilize less computational resources. Taxy [5] uses k-mer distribution in reference genomes and metagenomes and a mixture model to identify the organisms. RAIphy [6] uses k-mers to build relative abundance index, classification metric and the iterative algorithm to refine the model and estimate the abundance. Composition-based method have been proven to be efficient for the analysis of metagenomes, but its accuracy depends on the selection of informative reference genomes, which are used to find sequence characteristics. CLARK [7] uses target-specific or discriminative k-mers, which are genomic regions that uniquely characterize each genome. Then, reads are assigned to the genome based on the highest number of matches of the reads’ k-mers to a target-specific k-mer set.

Although the main objective of metagenomics analysis focuses on profiling known bacteria, it is complicated by the presence of unknown bacteria (or those without sequenced genomes). To the best of our knowledge, only MicrobeGPS [8] provides a basic analysis of unknown bacteria in how they are similar to known bacteria. It does not address the scenario where unknown bacterial genomes are vastly different from already-sequenced reference genomes.

To address this challenge, this work focuses on identifying and quantifying unknown bacteria in microbial communities. In this context, *unknown bacteria* are those whose genomes have not been sequenced. Given short reads from a microbial community that contain genomic materials from known and unknown bacteria, the method works by (i) first separating reads from known bacteria and unknown bacteria, and then (ii) clustering reads from unknown bacteria into multiple clusters; each cluster represents a hypothetical unknown bacterium. Importantly, the method utilizes only reads from 16S rRNA genes as a means to accomplish these tasks. Due to its high conservation, historically, the 16S rRNA gene has been used as a marker for taxonomic and phylogenetic analyses ([9, 10]). In the context of metagenomics, whose analyses depend on only short reads and not entire genes, the 16S rRNA gene was recently used as a means to construct functional profiles of microbial communities [11].

Using the 16S rRNA gene instead of whole genome information is not only computational efficient but also economical; Illumina indicated that targeted sequencing of a focused region of interest reduces sequencing costs and enables deep sequencing, compared to whole-genome sequencing. On the other hand, as observed by [8], by focusing exclusively on one gene, one might lose essential information for advanced analyses. We, however, will provide an analysis that demonstrates that at least in the context of oral microbial communities, the 16S rRNA gene retains sufficient information to allow us detect unknown bacteria.

## Methods

### Overview

Our method for identifying unknown bacteria from short reads that come from 16S rRNA genes of all bacteria (including known and unknown bacteria) in an environmental sample works as follows:

1. Reads are first roughly assigned to known bacteria. This is done by aligning those reads to the collection of already-sequenced 16S rRNA genes of known bacteria. The alignment process can be done using a good aligners such as Bowtie2 [12], BWA-MEM [13], Soap2 [14], RandAL [15]. We used Bowtie2 due to the efficiency and flexibility of the software package. The aligner works by creating an index $\mathcal {R}$ of reference 16S rRNA genes, which come from known (already-sequenced) bacterial genomes.
2. Reads that are not mapped to $\mathcal {R}$ are presumed to belong to 16S rRNA genes of unknown bacteria. We used SAMtools [10, 16] to collect unmapped reads from the results of Bowtie2. At this point, it is possible and actually expected that (i) some reads that belong to unknown bacteria have been mistakenly mapped to $\mathcal {R}$, and (ii) some reads that belong to the 16S rRNA gene of some known bacteria are mistakenly not mapped to $\mathcal {R}$. Thus, the set of unmapped reads, $\mathcal {U}$, contain both false positives and false negatives.
3. The unmapped reads, $\mathcal {U}$, are then clustered into distinct clusters. Each cluster represents a hypothetical unknown bacterium. An additional post-processing step can be applied to (i) remove clusters with too few reads as they do not possess sufficient information and (ii) split large clusters that might contain reads belong to more than one bacteria. At this point, it is possible that (i) multiple clusters can represent the same unknown bacterium and (ii) an unknown bacterium is not represented at all by any cluster. Both cases are not desirable and they both affect the accuracy of predicting the number of unknown bacteria.

### Uniqueness of the 16S rRNA gene in the human oral microbiome

Using the 16S rRNA gene as marker instead of the whole genome for identification and profiling bacterial communities potentially can lose a lot of information. On the other hand, this gene is highly conserved, which means that using it as the marker is more advantageous than using the whole genome since the reference gene in our database is less likely to be different than the gene in bacteria collected from environmental samples. Our analysis with a dataset that consists of 889 bacteria in the Human Oral Microbiome database suggests that the use of the 16S rRNA gene as marker is justified because there is a sufficient amount of information in this gene among different bacteria to help distinguish these bacteria. Consequently, the use of the 16S rRNA gene as marker to distinguish bacteria enjoys both the advantageous characteristics of the gene and having sufficient information required for the task.

To analyze the effectiveness of using the 16S rRNA gene as marker, we quantify the uniqueness of the gene among the set of 16S rRNA genes in bacteria of interest. To be precise, let *G*={*g*_1_,*g*_2_,⋯,*g*_*n*_} be the set of 16S rRNA genes of bacteria of interest. Define *U*(*k,g*_*i*_,*g*_*j*_) to be the number of k-mers in *g*_*i*_ that are not in *g*_*j*_ or $g_{j}^{rc}$ divided by |*g*_*i*_|−*k*+1, where $g_{j}^{rc}$ is the reverse complement of *g*_*j*_. Note that 0≤*U*(*k,g*_*i*_,*g*_*j*_)≤1. In particular, *U*(*k,g*_*i*_,*g*_*j*_) being 1 means that all k-mers in *g*_*i*_ do not occur in *g*_*j*_ or $g_{j}^{rc}$. Thus, when *U*(*k,g*_*i*_,*g*_*j*_)=1, it is likely that reads much longer than *k* coming from *g*_*i*_ will not be mistakenly mapped to *g*_*j*_. Further, for each *g*_*i*_, define

$$U(k, g_{i}) = \min_{1 \le j \le n, j \neq i} U(k, g_{i}, g_{j}) $$

Thus, the *uniqueness score*, *U*(*k,g*_*i*_), is a conservative measure of uniqueness of *g*_*i*_ in the whole set *G*. The closer *U*(*k,g*_*i*_) is to 1, the more unique it is, and the more likely that reads much longer than *k* from *g*_*i*_ will not be mistakenly mapped to any other gene *g*_*j*_ in *G*.

**Fig. 1:**
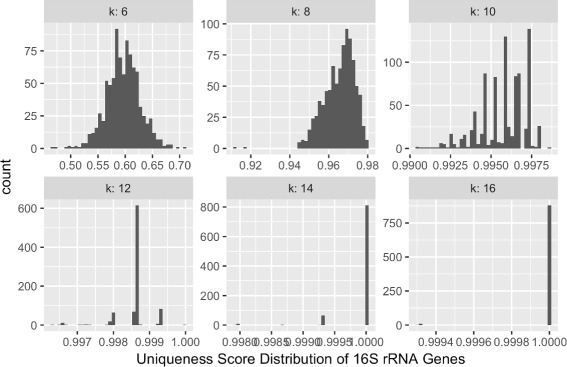
Distributions of *U*(*k,g*_*i*_) of 16S rRNA genes suggest that k-mers longer than 16 can effectively be used to distinguish bacteria in the human oral microbiome

Figure 1 shows, for different values of *k*, the distributions of *U*(*k,g*_*i*_) of 889 16S rRNA genes obtained from the Human Oral Microbiome database. We can see that the distribution of *U*(6,*k*_*i*_) peaks at around 0.58; i.e. around 88 genes have uniqueness scores at approximately 0.58. When *k*=8, most genes have uniqueness scores at around 0.97. When *k*=16, most genes have uniqueness scores at 1. When *k*≥18, we observed that all genes have uniqueness score of 1. This means for each gene in *G*, we can distinguish it with other genes using 18-mers. It also means that given reads produced by current technologies (e.g. ≥10), it is likely that reads that come from some gene *g*_*i*_ will not be mistakenly mapped to any gene other than *g*_*i*_.

### Clustering unmapped reads

**Fig. 2:**
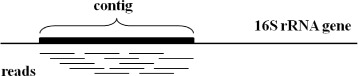
Reads mapped to a contiguous region of a 16S rRNA gene

The clustering procedure described in Step 3 of Section Overview is a critical component of this method. Technically, each cluster is a collection of reads that cover a contiguous genomic region. In other words, if one was to align these reads to the correct genomic region of a 16S rRNA that contains these reads, they would form a contiguous sequence. See Fig. 2.

We employ the data structure that is similar to a Union-Find data structure [17] to partition unmapped reads in $\mathcal {U}$ into a disjoint set of subsets. Each subset or cluster would represent a contiguous genomic region. This data structure *C* has following methods:

- MakeSet(*x*), which creates a singleton set containing the element *x*.
- Union(*x,y*), which unions the two disjoint sets that contain, respectively, *x* and *y*.
- Find(*x*), which finds the set that contains *x*.
- Clusters(), which returns all disjoint subsets that C maintains.

These methods can be encapsulated in data structure that is similar to the Union-Find data structure. Given the set of unmapped reads, $\mathcal {U}$, the clustering procedure (as described in Step 3, Section Overview) can be described in Algorithm 1, which is described in an inefficient manner to help understandability; our actual implementation is more efficient. Essentially, the procedure looks at all pairs of unmapped reads and – if they overlap – merges the contigs to which they belong. Since reads can be in either the primary or the complementary strand, the determination of overlapping of two reads must account for this fact. First, given two sequences, define *O*(*a,b*)=*HAM*(*pre*(*a,k*),*suf*(*b,k*)), where *pre*(*a,k*) is the *k*-prefix of *a*; *suf*(*b,k*) is the *k*-suffix of *b*; and HAM is the Hamming distance function. Then, the overlapping of two reads *x* and *y* is determined as follows: Overlap(*x,y*) is True and only if

$${\max(O(x,y), O(x^{rc},y), O(x,y^{rc}), O(x^{rc},y^{rc})) \over \min(|x|, |y|)} \ge \tau $$

where |*x*| is the length of *x*; *x* ^*rc*^ is the reverse complement of *x*; and *τ* is an empirically determined parameter.

### Post clustering processing

Clusters produced by Algorithm 1 are predicted raw representations of different bacteria. Additional processing can be done to improve prediction accuracy. In particular, two heuristics can be employed. First, clusters containing too few reads should be removed as they do not possess enough information to give sufficient confidence in prediction. Second, clusters with too many reads might contain reads that belong to more than one bacteria. We consider heuristics that decompose graphs into large disjoint clusters representing different bacteria. One of such heuristics is based on a well-studied problem in network analysis: decomposition of graphs into dense subgraphs [18]. To adopt this strategy, we represent the set of unmapped reads in cluster *i* as a graph, *G*_*i*_, in which vertices represent reads and edges represent overlapping of read pairs. Specifically, there is an edge (*u,v*), if and only if Overlap (*u,v*) is true. As defined in Section Clustering Unmapped Reads, the function Overlap examines the overlapping of reads as well their reverse complements. With this representation, reads within each cluster that belong to different bacteria tend to form dense subgraphs of *G*_*i*_. These subgraphs are connected with each other by edges that represent the overlapping of similar reads belonging to different bacteria.

### Method evaluation

As clusters returned by Algorithm 1 represent predicted species, the quality of prediction can be quantified in terms of how closely the clusters resemble the set of bacteria that reads belong to. Let *T*={*T*_1_,⋯,*T*_*n*_} be the set of bacteria that unmapped reads belong to and *C*={*C*_1_,⋯,*C*_*m*_} be the set of clusters that our method assigns the reads to. Although there are many different ways the accuracy of clusterings can be evaluated, we chose four different metrics that evaluate clustering quality in different meaningful and complementary ways.

Mutual information is an information-theoretic measure of how similar two joint distributions are. In the context of clustering, the mutual information between two clusterings *T* and *C* is defined as

$$MI(T, C) = \sum\limits_{i=1}^{n} \sum\limits_{j=1}^{m} P(i,j) \log {P(i,j) \over P(i) P(j)} $$

where *P*(*i,j*) is the probability that a read belongs to both *T*_*i*_ and *C*_*j*_; *P*(*i*) is the probability that a read belongs to *T*_*i*_; *P*(*j*) is the probability that a read belongs to *C*_*j*_. The Adjusted Mutual Information (**AMI**) [19] of two clusterings is an adjustment of mutual information to account for chance and is defined as follows:

$$AMI(T, C)={MI(T, C) - E(MI(T, C)) \over \max(H(T), H(C)) - E(MI(T, C))} $$

where *E*(*MI*(*T,C*)) is the expected mutual information of two random clusterings and *H*(*T*) is the entropy of the clustering *T*. An AMI value of 0 occurs when the two clusterings are random, whereas a value of 1 occurs when *C* and *T* are identical.

Rand Index is a common measure in classification problems, where the measure takes into account directly the number of correctly and incorrectly classified items.

$$ RI(T,C) = {2(a+b) \over n(n-1)} $$

where *a* is the number of pairs of reads that are in the same cluster in *T* and *C*; and *b* is the number of pairs of reads that are in different clusters in *T* and *C*. The Adjusted Rand Index (**ARI**) was introduced to take into account when the Rand Index of two random clusterings is not a constant value [20]. An ARI value of 0 occurs when two *C* and *T* are independent, whereas a value of 1 means *C* and *T* are identical.

In addition to AMI and ARI, we also considered two complementary metrics, introduced by [21]: **homogeneity** and **completeness**. A clustering is homogenous if each cluster *C*_*j*_ contains only reads that come from some bacterium *T*_*i*_. A clustering is complete if all reads that belong to any bacterium *T*_*i*_ are placed into some cluster *C*_*j*_. These two metrics are opposing in that it is often hard to achieve high scores on both homogeneity and completeness. A few examples might help understand this intuition:

- *T*=*C* if and only if both homogeneity are completeness scores are 1. *T* being identical to *C* only occurs when reads in each *T*_*i*_ are placed in exactly one *C*_*j*_, and all reads in each *C*_*j*_ come only from one *T*_*i*_.
- Suppose *T*={{*r*_1_,*r*_2_},{*r*_3_,*r*_4_}} and *C* = {{*r*_1_,*r*_2_,*r*_3_,*r*_4_}}. Then, the completeness score is 1, because all reads that belong to *T*_1_ (and respectively to *T*_2_) are placed in the same cluster in *C*. On the other hand, the homogeneity score is 0, because reads in the only cluster in *C* come from different bacteria in *T*.
- Suppose *T*={{*r*_1_,*r*_2_},{*r*_3_,*r*_4_}} and *C*={{*r*_1_,*r*_3_},{*r*_2_,*r*_4_}}. Then, both completeness and homogeneity scores are 0.

## Results and discussion

In this section, we report experimental results that show various aspects of accuracy and robustness of this method. Accuracy is measured by four different metrics Adjusted Mutual Information (AMI), Adjusted Rand Index (ARI), Homogeneity and Completeness.

### Mock microbial communities

Experiments were conducted on 16S rRNA genes obtained from 889 sequences cataloged by the Human Oral Microbiome Database. The lengths of genes vary between 1,323 to 1,656 bases. We simulated mock microbial communities at various settings in order to be able to compare ground truths and predicted values and ascertain the accuracy of the method. Each mock community consists of (A) *known* bacteria, whose 16S rRNA genes were used to filter out known bacteria, and (B) *unknown* bacteria, whose 16S rRNA genes must be identified and separated into different clusters representing different unknown bacteria.

These mock communities were synthetically created to evaluate various aspects of our method. In our experiments, short reads from 16S rRNA genes were generated using Grinder [22] using parameters for the Illumina sequencing platform. Mean read length was 150 with a standard deviation of 20. Read coverage was between 10x to 100x and the percentage of unknown bacteria varied from 1 to 16%. To study how one parameter affects the accuracy of the method, we used mock communities in which only that parameter varied while the others were kept constant.

### The affect of coverage on prediction accuracy

First, we examined how the method’s accuracy (in terms of completeness, homogeneity, mutual information and Rand index) varied at increasing read coverages. We expected that having more reads means having more information and that would result in an observed increase in accuracy. In this experiment, read coverage in mock communities varied from 10x to 100x. The percentage of unknown bacteria in these communities were kept constant at 8%.

**Fig. 3:**
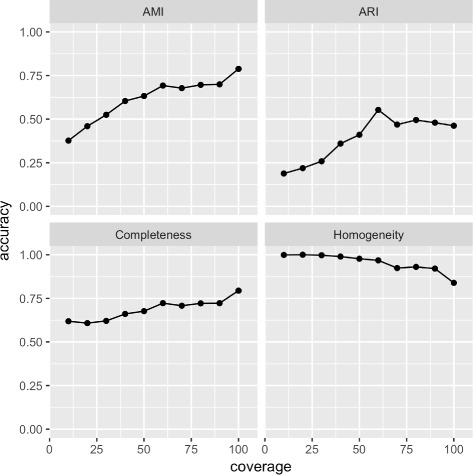
Accuracy of predicting unknown bacteria (measured by 4 different metrics) at read coverage ranging from 10x to 100x

Figure 3 shows accuracies measured by 4 different metrics. As expected, prediction accuracy was higher at higher coverage for 3 of the measures. Additionally, accuracy values measured by AMI are generally higher than ARI. AMI tells us about the degree of randomness of a predicted clustering compared to the ground-truth clustering, whereas ARI attempts to quantify the item pairs that are in the same and different subsets. Our interpretation of this observation is that while predictions are not random, there are still structural information among clusters or within clusters that our method has not fully exploited.

Further, predictions were homogeneous than complete. This means that (i) a cluster more likely contains only reads that belong to some bacterium, and (ii) reads belonging to a bacterium could be placed in multiple clusters. Observation (i) confirmed that the method worked as it should. To understand observation (ii), note that if reads belonging to a gene do not assemble into a contiguous sequence (due to low or non-uniformity of coverage), then reads belonging to the gene will be placed into multiple clusters.

Finally, as coverage approached 100x, clusters became less homogenous. This happened because having more reads increased the change of mistakenly placing reads into clusters representing different bacteria. In this experiment, 80x appears to be a good coverage.

### The affect of unknown bacteria concentration

To study the affect of the amount of unknown bacteria has on prediction accuracy, we evaluated our method with mock communities in which percentage of unknown bacteria varied from 2 to 16%, while read coverage was kept constant at 40x with 10 random replicates at each percentage.

**Fig. 4:**
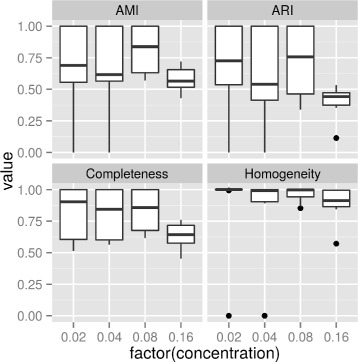
Accuracy of predicting unknown bacteria (measured by 4 different metrics) at different amount of unknown bacteria

The result of this experiment is summarized in the box plot in Fig. 4. As expected, prediction accuracy (as measured by AMI, ARI and Completeness) tended to decrease with more unknown bacteria. On the other hand, homogeneity were not effected very much. The result shows that accuracy starts dropping dramatically when the concentration of unknown bacteria reaches 16%. We hope that future improvements can increase this number.

## Conclusions

Although it is known that 16S rRNA genes can be used to distinguish known bacteria, we demonstrated that only *reads* from these genes can be used to predict the number of unknown bacteria in oral microbial communities. Advantages include (i) a reduction in cost and computational processing, and (ii) the high conservation of 16S rRNA genes increases the chance of reference genetic materials being highly similar to those of bacteria in environments, which eliminates multiple sources of errors and challenges.
